# Impact of Cypermethrin on Fingerlings of Common Edible Carp *(Labeo rohita)*

**DOI:** 10.1100/2012/291395

**Published:** 2012-04-30

**Authors:** Sudhanshu Tiwari, Richa Tiwari, Ajay Singh

**Affiliations:** ^1^Natural Product Laboratory, Department of Zoology, D.D.U. Gorakhpur University, Gorakhpur 273009, India; ^2^Department of Botany, D.D.U. Gorakhpur University, Gorakhpur 273009, India

## Abstract

Laboratory evaluations were made to assess the toxicological and biochemical effect of cypermethrin on fingerlings of common edible freshwater culture carp *(Labeo rohita)*. There was a significant negative (*P* < 0.05) correlation observed between effective doses of cypermethrin and exposure periods; that is, LC_50_ values decreased from 0.323 *μ*g/L (6 h) to  > 0.278 *μ*g/L (12 h),  > 0.240 *μ*g/L (18 h) and >0.205 *μ*g/L (24 h). Exposure to sublethal doses of cypermethrin for 24 h and 96 h exposure period caused significant (*P* < 0.05) time- and dose-dependent alterations in total protein, total free amino acids, nucleic acids, glycogen, pyruvate, and lactate level and in the activity of enzyme protease, alanine aminotransferase, aspartate aminotransferase, acid phosphatases, alkaline phosphatases, acetylcholinesterase, and cytochrome oxidase in liver and muscle tissues of fish. Thus, cypermethrin has potent piscicidal activity against fingerlings of fish *Labeo rohita* and adversely affects their behavioural patterns, shifting aerobic pathway of fish respiration towards anaerobic pathway and also inhibiting energy production by suppressing ATP synthesis.

## 1. Introduction

Synthetic pyrethroids are more effective than other insecticides like carbamate and organophosphatase against several pests [1]. Besides controlling hazardous insect pest population synthetic pyrethroids also affect several nontarget organisms [2].

Cypermethrin, a synthetic pyrethroid is a broad-spectrum insecticide, used extensively in households and industrial and agriculture fields [3] for control of several insect pests [4]. Due to indiscriminate use, cypermethrin makes their entrance into natural water bodies through agriculture run-off and ultimately affects the several nontarget aquatic organisms; for instance, it inhibits growth and several metabolic activities of crustaceans [5], adversely affects fish metabolism [6] and hematology [7], and thus adversely affects fish meat quality and fish population [8]. Cypermethrin is also highly toxic against freshwater snail *L. accuminata *and also affects both their metabolism and reproduction [9, 10].

Srivastava and Kaushik [11] reported that the pesticides accumulating in the vital organs, such as liver and muscle, cause organ dysfunction, culminating in death of fishes. Certain enzymes are essential for metabolic functions, disturbance of enzyme activity in fish serves as early indicator of toxicant impact [12]. Hence, the present work was designed to assess the toxic effect of cypermethrin on fingerlings of *Labeo rohita* and its sublethal effects on its metabolism. *Labeo rohita* is common, freshwater, edible fish of capture fishery not only in India but also elsewhere, due to their high nutritive food value.

## 2. Materials and Methods

### 2.1. Collection and Maintenance of Experimental Animal

Healthy, fresh fingerlings of fish *Labeo rohita* (6.0 ± 1.5 cm in total length, 8.0 ± 2.0 gm in weight) were obtained from the Gorakhnath Government Hatchery Center, Chapia, Gorakhpur, Utter Pradesh, India. The collected fishes were maintained in glass aquaria containing 100 L dechlorinated tap water for seven days to acclimatize under laboratory conditions. The aquarium water was aerated continuously, and food was provided in the form of dried, small pellets of Tokyo, special fish food produced in Japan. Water was changed at every 24 h. Experimental water conditions were atmospheric temperature 31.5–32.5°C; water temperature 28.5–30.0°C; pH 7.2–7.5; dissolved oxygen 7.7–8.2 mg/L; free carbon dioxide 4.5–5.5 mg/L; bicarbonate alkalinity 104.5–106.5 mg/L [13].

Cypermethrin ([*S, R*]-*N*-*α*-cyno-3-phenoxybenzyl-(IR, IS, *cis, trans*)-2, 2-dimethyl-3, (2, 2-dichlorovinyl) cylcopropane carboxylate) manufactured by M/S Tropical Agrosystem Pvt. Ltd. Chennai, India, and purchased from local market of Gorakhpur, Uttar Pradesh, India.

### 2.2. Toxicity Assay

Toxicity experiment was performed by the method of Singh and Agarwal [14]. The fingerlings of *L. rohita* were exposed at four different concentrations, of cypermethrin. Six aquaria were set up for each concentration and each aquarium contains ten fishes in 10 L dechlorinated tap water. Control animals were kept in same condition without any treatment. Mortality was recorded at every 6 h up to 24 h exposure period. Fishes were considered dead if they failed to respond to stimulus provided with glass rod. Lethal concentrations, upper and lower confidence limits, slope value, “*t*” ratio, “*g*” factor, and heterogeneity were calculated through probit log analysis method by using POLO computer programme of Russel et al. [15].

### 2.3. Biochemical Analysis

Ten fishes were kept in glass aquaria containing 10 L dechlorinated tap water and exposed for 24 h and 96 h exposure periods at sublethal doses (40% and 80% of 6 h and 24 h LC_50_) of cypermethrin. Control animals were held in same condition without any treatment. After completion of treatment, the test animals were removed from aquaria, washed with water, and killed. After having the killed animals, the liver and muscle tissue were quickly dissected out, freed from adipose and connective tissues, frozen in liquid nitrogen, and stored at −70°C, which was used for biochemical analysis.

### 2.4. Methods by Which Biochemical Parameters Were Measured 

#### 2.4.1. Total Protein

Total protein level was estimated by the method of Lowry et al. [16]. Homogenates (5 mg/mL) were prepared in 10% trichloroacetic acid (TCA). Bovine serum albumin was used as standard. Result was expressed as *μ*g/mg.

#### 2.4.2. Total Free Amino Acids

Total free amino acid level was estimated by the method of Spices [17]. Homogenates (10 mg/mL) were prepared in 95% ethanol. Glycine were used as standard. Result was expressed as *μ*g/mg.

#### 2.4.3. Nucleic Acids

Nucleic acids (DNA and RNA) were estimated by the methods of Schneider [18]. Homogenates (10 mg/mL) were prepared in 5% TCA. Calf thymus DNA and yeast RNA was used as standard for DNA and RNA, respectively. Result was expressed as *μ*g/mg.

#### 2.4.4. Glycogen

Glycogen level was estimated by the method of Van der Vies [19]. Homogenate (10 mg/mL) was prepared in 5% TCA. Glucose was used as standard. Result was expressed as *μ*g/gm.

#### 2.4.5. Pyruvate

Pyruvate level was estimated by the method of Friedemann and Haugen [20]. Homogenate (50 mg/mL) was prepared in 10% TCA. Sodium pyruvate was used as standard. Result was expressed as *μ*g/mg.

#### 2.4.6. Lactate

Lactate level was estimated by the method of Huckabee [21]. Homogenate (50 mg/mL) was prepared in 10% TCA. Sodium lactate was used as standard. Result was expressed as *μ*g/mg.

### 2.5. Enzymological Analysis

#### 2.5.1. Protease

Protease enzyme activity was estimated by the method of Moore and Stein [22]. Homogenate (50 mg/mL) was prepared in cold distilled water. Tyrosine was used as standard. Result was expressed as *μ* moles tyrosine/mg protein/h.

#### 2.5.2. Alanine Aminotransferase (ALAT) and Aspartate Aminotransferase (AAT)

ALAT and AAT enzyme activities were estimated by the method of Reitman and Frankel [23]. Homogenates (50 mg/mL) were prepared in 0.25 M cold sucrose solution. Oxaloacetic acid was used as standard. Result was expressed as *μ* moles pyruvate/mg protein/h.

#### 2.5.3. Acid (AC) and Alkaline (AK) Phosphatase

AC and AK activities were determined according to the method of Andersch and Szcypinski [24]. Homogenates (50 mg/mL) were prepared in ice-cold 0.9% sodium chloride solution. *ρ*-nitro phenol was used as standard. Result was expressed as *ρ*-nitro phenol formed/30 min/mg protein.

#### 2.5.4. Acetylcholinesterase (AChE)

AChE enzyme activity was estimated by the method of Ellman et al. [25]. Homogenate (50 mg/mL) was prepared in 0.1 M phosphate buffer, pH 8.0, for 5 min in an ice bath. Glutathionine was used as standard. Result was expressed as *μ* mol “SH”hydrolysed/min/mg protein.

#### 2.5.5. Cytochrome Oxidase (CyO)

CyO enzyme activity was estimated by the method of Cooperstein and Lazarow [26]. Homogenate (50 mg/mL) was prepared in 0.33 M phosphate buffer, pH 7.4, for 5 min in ice bath. Cytochrome-C was used as standard. Result was expressed as arbitrary units/min/mg proteins.

Each assay was replicated at least six times, and the values have been expressed as mean ± SE of six replicates. Student's “*t*” test was applied to locate significant difference with controls [27]; two-way analysis of variance (ANOVA) and least significant difference (LSD) test were also applied for appropriate statistical analysis [28] for interpretation.

## 3. Results

### 3.1. Behavioural Changes

Exposures to 0.176, 0.264, 0.352, and 0.440 *μ*g/L cypermethrin caused significant visible behavioural changes in the fingerlings of *L. rohita*. Immediately after treatment, fish show body irritation and feel suffocation, and they came at the water surface for gasping the air. The initial 30 minutes was a period of hyperactivity, during which fish become restless. After 30–60 minutes, their swimming activities were slowed down and irregular, jerky movements and loss of body equilibrium were observed. After some time, they try to stay at upper water surface and loss of body equilibrium was pronounced. Finally their entire activity decreases, and they settled down at the base of water aquaria and died. Fishes of control group were free from such behavioral changes.

### 3.2. Toxicity Experiment

Table 1 shows that fish mortality was both time as well as dose dependent, and there was a significant (*P* < 0.05) negative correlation between effective doses and exposure periods. Thus increasing exposure period, LC_50_ values decreased from 0.323 *μ*g/L (6 h) to > 0.278 *μ*g/L (12 h), > 0.240 *μ*g/L (18 h), and > 0.205 *μ*g/L (24 h). A similar trend was also observed in case of LC_10_ and LC_90_ values (Table 1).

The slope values of toxicity data (Table 1) were steep, and heterogeneity factor was less than 1. The regression test (“*t*” ratio) was greater than 1.96, and the potency estimation test (“*g*” value) was less than 0.5 at all probability levels.

### 3.3. Biochemical Effects

Exposure to sublethal doses of cypermethrin of 0.129 *μ*g/L, 0.258 *μ*g/L for 24 h and 0.082 *μ*g/L, 0.164 *μ*g/L for 96 h exposure period caused significant (*P* < 0.05) alterations in protein as well as carbohydrate metabolism of the fingerlings of fish *L. rohita* in both liver and muscle tissues (Tables 2 and 3).

Total protein, nucleic acids (DNA and RNA), glycogen, and pyruvate level were significantly reduced (*P* < 0.05) while total free amino acid and lactate level were significantly enhanced (*P* < 0.05) in liver and muscle tissues after exposure to sublethal doses (Tables 2 and 3).

### 3.4. Enzymological Analysis

Enzymological analysis clearly demonstrated that activities of enzyme acid, alkaline phosphatase, AChE, and CyO were significantly (*P* < 0.05) reduced while activities of enzyme proteases, ALAT, AAT enzyme activity, were significantly enhanced (*P* < 0.05) in liver and muscle tissues of fish after exposure to sublethal doses (Tables 2 and 3).

## 4. Discussion

Changed behavioral responses can be taken as index of the stress felt by the fish exposed to cypermethrin, by which they try to reduce excess entry of cypermethrin present in the medium or minimize damage to their body tissues. Similar behavioral changes were also observed in guppy fish *Poecilia reticulata*, after exposure to cypermethrin [3, 5] and permethrin [29]. Fishes of control group are free from such behavioural changes, which indicates cypermethrin was responsible for above altered behaviour and fish mortality. Animal behaviour is usually regulated by neurosecretion such as AChE, at the synapse [30]. From Result, it is evident that cypermethrin inhibits activity of enzyme AChE, this enzyme is present in synaptic regions and mediates transmission of impulses by breaking acetylcholine into acetic acid and choline. The acetylcholine at neural and neuromotor regions upon accumulation causes “hyperexcitability,” which in turn might also influence behavioural pattern and may lead to death of fish [30]. Das and Mukherjee [31] also reported that cypermethrin inhibits AChE activity in the brain of *L. rohita* fingerlings. Cypermethrin acts as neuroactive poisons, interfering with the nerve impulse conduction and changes the nerve membrane permeability [30].

Similar results were also observed in organophosphate- and carbamate-pesticide-exposed fishes, due to inhibition of enzyme acetylcholinesterase activity [32].

The positive correlation between cypermethrin dose and fish mortality may be due to increased concentration of cypermethrin in test aquaria resulting in their increased entrance inside fish bodies.

The steep slope values indicate that increased fish mortality occurred with relatively small increase in cypermethrin dose. “*t*” ratio is greater than 1.96, which shows that regression is significant. Values of heterogeneity factor less than 1.0 denote that in the replicate tests of random samples the concentration response lines would fall within 95% confidence limits, and thus the model fits the data adequately. The index of significance of potency estimation “*g*” indicates that the value of the mean is within the limits at all probability levels as it is less than 0.5 [33].

The altered behavioral symptoms show metabolic changes in the biochemical parameters particularly related to carbohydrate and protein metabolism [34]. Cypermethrin induces stress condition result in less availability of oxygen, in turn less ATP production in tissues and thus adversely affecting oxidative metabolism [34]. During stress condition, fishes needed more energy to detoxify the toxicants and to overcome stress. And by this they try to minimize the toxic effect of cypermethrin. Carbohydrates are the primary and immediate source of energy. In stress condition, carbohydrates reserve depleted to meet energy demand. Depletion of glycogen may be due to direct utilization for energy generation, a demand caused by cypermethrin-induced hypoxia [10].

Carbohydrate metabolism is mainly concerned with fulfilling energy demand of animals by its anaerobic segment or glycolysis in which the break down of glucose or glycogen through the embden-meyerhof pathway occurs, and aerobic segment induces oxidation of pyruvate to acetyl co-A to be utilized through citric acid cycle [35]. The pesticides inhibit energy synthesis by suppressing aerobic oxidation of carbohydrate leading to energy crisis in animals [10].

The level of tissue lactate content acts as an index of anaerobiosis, which might be beneficial for animal to bear hypoxic condition whereas pyruvate level in tissue can be taken as a measure of aerobic condition. Under stress condition, with the increases of lactate content, there was a decrease in pyruvate content, which suggests a shift towards anaerobiosis as a consequence of hypoxia, leading to respiratory distress [30]. The lactic acid accumulation in muscle is an augmentation of glycolytic pathway as a consequence of stress. The prevalence of hypoxia during cypermethrin treatment should have led to respiratory distress when it is forced to depend on deriving energy by anaerobiosis. Increase in lactic acid level in fingerlings of *L. rohita* suggests a possibility of flocculation of cypermethrin (i.e., layering over gill membranes and filaments) thus altering the aerobic nature and causing a shift towards anaerobiosis. There are many reports suggesting accumulation of lactate due to increase in the glycolytic segment induced by cypermethrin insecticide [36]. The increase in tissue lactate content may be due to its involvement in osmoregulation. During stress condition, there was a decrease in osmotic regulation of internal body media of animal by loss of mono-as well as divalent cations which is compensated by the animal with the increase of organic ions like lactate, amino acid, and so forth [30]. The decrease of pyruvate level may be due to its conversion to lactate or due to its mobilization to form amino acids, lipids, and so forth, addition to its role as a detoxification factor [30]. Thus it may be presumed that there is a tendency to shift the aerobic pathway to anaerobic pathway of fish respiration, to meet energy demands for the physiological and metabolic activities augmented by stress induced by cypermethrin.

Since fish have a very little amount of carbohydrates [30], the next alternative source of energy to meet the increased energy demand is protein. The decrease in protein level in liver and muscle tissues may be a result of trying to meet higher energy demands for metabolic purposes. Increase in level of free amino acids is due to breakdown of protein and also due to impaired incorporation of amino acids in protein synthesis and decline in nucleic acid level [37]. Similar result was also observed in *Cyprinus carpio* fish after sublethal exposure of cypermethrin [38]. The decreases in total protein level and increases in total free amino acid level suggest the high protein hydrolytic activity is due to elevation of protease enzyme activity.

Aspartate and alanine aminotransferase function as a link between carbohydrate and protein metabolism [39]. Under exposure of cypermethrin, the activity of both aminotransferases was highly elevated in all the tissues of fish [38], confirming the augmentation of stress as a consequence of exposure of cypermethrin. Such a situation takes place during present study. To complete the energy demand during stress condition, the carbohydrate and its precursors are increased to maintain carbohydrate metabolism at sustained levels. It is likely that this stress might be the result of augmentation of energy demand during the toxicity period where the depletion in energy resources is high. Since the amino acid level also increased (Tables 2 and 3), it is evident that both ALAT and AAT activities are being stepped up to be in line with the increasing energy demands. Both in liver and muscle tissue, ALAT predominates over AAT where the feeding of amino acids into energy cycle is more through alanine-pyruvate pathway representing anaerobic tendency of the tissues.

Cytochrome oxidase transfers electrons to their final acceptor oxygen in electron transfer system (ETS). It produces ATP molecules thereby influencing other cellular metabolic process. Sublethal experiment with cypermethrin causes decrease in cytochrome oxidase activity in liver and muscle tissues, which shows a profound impact on the oxidative metabolism [30]. Decrease in cytochrome oxidase activity either due to reduced availability of oxygen, which in turn has reduced the capacity of the ETS to produce ATP molecules, or because after metabolism of cypermethrin, cyano group separated and attached with metal ion (Fe, Cu) present in cytochrome oxidase enzyme and reduced their activity [40]. Tripathi and Singh [34] also reported that cypermethrin inhibits cytochrome oxidase enzyme activity in different body tissues of freshwater snail *L. acuminata* and thus reduces ATP synthesis, same as the experiments conducted by Kakko et al. [3].

## 5. Conclusion

At last, we can conclude that cypermethrin has potent piscicidal activity against edible freshwater fish *L. rohita* fingerlings, and its ultimate mode of action is respiratory pathway of fish, which shifts towards anaerobic segments and adversely affects their oxidative metabolism and inhibits energy production by suppressing ATP synthesis. Thus present study calls for careful application of cypermethrin in insect-controlling operations, lest it should pollute the inland waters threatening the life of fish and other aquatic organisms.

## Figures and Tables

**Table 1:** Toxicity of cypermethrin against fingerlings of fish *Labeo rohita*.

Exposure periods	Effective dose (LC values, *μ*g/L)	Limits (*μ*g/L)		Slope value	“*g*” factor	“*t*” ratio	Heterogeneity
		LCL	UCL				
6 h	(i) LC_10_ = 0.203	0.173	0.225	6.33 ± 0.77	0.056	8.27	0.34
	(ii) LC_50_ = 0.323	0.302	0.347				
	(iii) LC_90_ = 0.515	0.461	0.610				

12 h	(i) LC_10_ = 0.189	0.166	0.207	7.64 ± 0.80	0.042	9.52	0.32
	(ii) LC_50_ = 0.278	0.261	0.295				
	(iii) LC_90_ = 0.409	0.378	0.457				

18 h	(i) LC_10_ = 0.172	0.152	0.187	8.92 ± 0.94	0.043	9.46	0.29
	(ii) LC_50_ = 0.240	0.225	0.254				
	(iii) LC_90_ = 0.334	0.311	0.367				

24 h	(i) LC_10_ = 0.154	0.136	0.168	8.41 ± 0.29	0.059	8.05	0.13
	(ii) LC_50_ = 0.205	0.193	0.217				
	(iii) LC_90_ = 0.273	0.254	0.301				

There was no mortality in control groups.

Batches of ten fishes were exposed to different concentrations of cypermethrin.

Concentrations given are the final concentrations (w/v) in aquarium water.

Regression coefficient showed that there was significant (*P* < 0.05) negative correlation between exposure periods and different LC values.

LCL: lower confidence limit; UCL: upper confidence limit.

**Table 2:** Exposure of fish *Labeo rohita *fingerlings to cypermethrin for 24 h exposure periods.

Parameters	Tissues	Control	Doses	
			0.129 *μ*g/L (40% of 6 h LC_50_)	0.258 *μ*g/L (80% of 6 h LC_50_)
TP	Liver	120.10 ± 0.10 (100)	108.09 ± 0.12 (90)^abc^	96.08 ± 0.14 (80)^abc^
	Muscle	130.00 ± 0.22 (100)	114.40 ± 0.14 (88)^abc^	94.90 ± 0.22 (73)^abc^
FAA	Liver	7.10 ± 0.30 (100)	8.52 ± 0.19 (120)^bc^	9.94 ± 0.14 (140)^abc^
	Muscle	13.25 ± 0.20 (100)	17.88 ± 0.19 (135)^abc^	20.54 ± 0.13 (155)^abc^
DNA	Liver	35.40 ± 0.22 (100)	31.86 ± 0.13 (90)^abc^	28.32 ± 0.12 (80)^abc^
	Muscle	3.20 ± 0.36 (100)	27.56 ± 0.22 (83)^abc^	23.24 ± 0.22 (70)^abc^
RNA	Liver	37.44 ± 0.33 (100)	29.95 ± 0.41 (80)^abc^	25.83 ± 0.21 (69)^abc^
	Muscle	38.35 ± 0.15 (100)	25.69 ± 0.16 (67)^abc^	20.33 ± 0.23 (53)^abc^
GY	Liver	2.10 ± 0.05 (100)	2.00 ± 0.06 (95)^c^	1.70 ± 0.01 (81)^abc^
	Muscle	1.70 ± 0.02 (100)	1.53 ± 0.05 (90)^abc^	1.34 ± 0.05 (79)^abc^
PY	Liver	1.99 ± 0.02 (100)	1.39 ± 0.06 (70)^ab^	1.27 ± 0.08 (64)^ab^
	Muscle	1.50 ± 0.01 (100)	1.16 ± 0.02 (77)^ab^	0.98 ± 0.04 (65)^ab^
LA	Liver	1.44 ± 0.03 (100)	1.58 ± 0.09 (110)^bc^	1.87 ± 0.03 (130)^abc^
	Muscle	1.25 ± 0.01 (100)	1.44 ± 0.03 (115)^abc^	1.81 ± 0.05 (145)^abc^
PR	Liver	0.4630 ± 0.01 (100)	0.5325 ± 0.02 (115)^bc^	0.6019 ± 0.06 (130)^abc^
	Muscle	0.3340 ± 0.04 (100)	0.4008 ± 0.06 (120)^bc^	0.5010 ± 0.02 (150)^abc^
AC	Liver	0.135 ± 0.006 (100)	0.119 ± 0.002 (88)^abc^	0.095 ± 0.004 (70)^abc^
	Muscle	0.131 ± 0.004 (100)	0.093 ± 0.003 (71)^abc^	0.077 ± 0.008 (59)^abc^
AK	Liver	0.479 ± 0.007 (100)	0.431 ± 0.004 (90)^abc^	0.383 ± 0.003 (80)^abc^
	Muscle	0.465 ± 0.009 (100)	0.381 ± 0.006 (82)^abc^	0.330 ± 0.003 (71)^abc^
ALAT	Liver	3.46 ± 0.30 (100)	4.22 ± 0.22 (122)^abc^	4.91 ± 0.15 (142)^abc^
	Muscle	2.76 ± 0.11 (100)	3.31 ± 0.10 (120)^abc^	4.14 ± 0.13 (150)^abc^
AAT	Liver	1.80 ± 0.09 (100)	2.07 ± 0.07 (115)^bc^	2.43 ± 0.08 (135)^abc^
	Muscle	1.44 ± 0.08 (100)	1.58 ± 0.06 (110)^bc^	2.10 ± 0.05 (146)^abc^
AChE	Liver	0.170 ± 0.03 (100)	0.094 ± 0.02 (55)^abc^	0.068 ± 0.01 (40)^abc^
	Muscle	0.190 ± 0.04 (100)	0.114 ± 0.03 (60)^ab^	0.082 ± 0.01 (43)^ab^
CyO	Liver	35.30 ± 0.20 (100)	28.24 ± 0.10 (80)^abc^	24.71 ± 0.10 (70)^abc^
	Muscle	33.44 ± 0.40 (100)	25.08 ± 0.23 (75)^abc^	21.07 ± 0.12 (63)^abc^

Values were mean ± SE of six replicates.

Values in parentheses were % change with control taken as 100%.

Student's “*t*” test shows ^a^significant value (*P* < 0.05), when treated groups were compared with controls.

ANOVA and LSD test show significant (*P* < 0.05), when ^b^treated groups were compared with controls and ^c^treated groups were compared with each other.

Total protein (TP), total free amino acids (FAA), pyruvate (PY), lactate (LA), glycogen (GY), protease (PR), acid phosphatase (AC), alkaline phosphatase (AK), alanine aminotransferase (ALAT), aspartate aminotransferase (AAT), acetylcholinesterase (AChE), and cytochrome oxidase (CyO).

**Table 3:** Results of exposure of fish *Labeo rohita *fingerlings to cypermethrin for 96 h exposure periods.

Parameters	Tissues	Control	Doses	
			0.082 *μ*g/L (40% of 24 h LC_50_)	0.164 *μ*g/L (80% of 24 h LC_50_)
TP	Liver	118.15 ± 0.30 (100)	88.61 ± 0.12 (75)^abc^	68.53 ± 0.13 (58)^abc^
	Muscle	127.05 ± 0.28 (100)	88.94 ± 0.17 (70)^abc^	64.80 ± 0.22 (51)^abc^
FAA	Liver	7.05 ± 0.30 (100)	9.87 ± 0.17 (140)^abc^	13.40 ± 0.27 (190)^abc^
	Muscle	12.90 ± 0.10 (100)	20.64 ± 0.15 (160)^abc^	27.09 ± 0.19 (210)^abc^
DNA	Liver	35.30 ± 0.12 (100)	29.30 ± 0.16 (83)^abc^	23.65 ± 0.12 (67)^abc^
	Muscle	33.20 ± 0.35 (100)	23.24 ± 0.22 (70)^abc^	18.26 ± 0.16 (55)^abc^
RNA	Liver	36.80 ± 0.73 (100)	27.60 ± 0.24 (75)^abc^	22.08 ± 0.21 (60)^abc^
	Muscle	37.20 ± 0.28 (100)	23.44 ± 0.18 (63)^abc^	18.97 ± 0.12 (51)^abc^
GY	Liver	2.04 ± 0.07 (100)	1.66 ± 0.03 (83)^abc^	1.42 ± 0.05 (71)^abc^
	Muscle	1.65 ± 0.09 (100)	1.32 ± 0.04 (80)^abc^	0.99 ± 0.03 (60)^abc^
PY	Liver	1.99 ± 0.02 (100)	1.00 ± 0.01 (50)^abc^	0.88 ± 0.06 (44)^abc^
	Muscle	1.50 ± 0.01 (100)	0.84 ± 0.03 (56)^abc^	0.78 ± 0.05 (52)^abc^
LA	Liver	1.44 ± 0.03 (100)	2.30 ± 0.08 (160)^abc^	2.65 ± 0.07 (184)^abc^
	Muscle	1.25 ± 0.01 (100)	2.19 ± 0.04 (175)^ab^	2.38 ± 0.07 (190)^ab^
PR	Liver	0.4610 ± 0.06 (100)	0.6224 ± 0.06 (135)^abc^	0.8529 ± 0.03 (185)^abc^
	Muscle	0.3290 ± 0.09 (100)	0.4738 ± 0.07 (144)^abc^	0.6580 ± 0.03 (200)^abc^
AC	Liver	0.133 ± 0.003 (100)	0.093 ± 0.05 (70)^abc^	0.077 ± 0.03 (58)^abc^
	Muscle	0.130 ± 0.001 (100)	0.082 ± 0.05 (63)^abc^	0.065 ± 0.04 (50)^abc^
AK	Liver	0.473 ± 0.004 (100)	0.388 ± 0.05 (82)^abc^	0.311 ± 0.07 (70)^abc^
	Muscle	0.455 ± 0.003 (100)	0.332 ± 0.04 (73)^abc^	0.273 ± 0.02 (60)^abc^
ALAT	Liver	3.46 ± 0.30 (100)	5.54 ± 0.17 (160)^abc^	6.23 ± 0.11 (180)^abc^
	Muscle	2.76 ± 0.11 (100)	4.58 ± 0.11 (166)^abc^	5.24 ± 0.12 (190)^abc^
AAT	Liver	1.80 ± 0.09 (100)	2.61 ± 0.08 (145)^abc^	3.11 ± 0.06 (173)^abc^
	Muscle	1.44 ± 0.08 (100)	2.23 ± 0.08 (155)^abc^	2.59 ± 0.03 (180)^abc^
AchE	Liver	0.170 ± 0.03 (100)	0.060 ± 0.01 (35)^abc^	0.037 ± 0.03 (22)^abc^
	Muscle	0.190 ± 0.04 (100)	0.068 ± 0.02 (36)^abc^	0.038 ± 0.05 (20)^abc^
CyO	Liver	34.60 ± 0.20 (100)	24.22 ± 0.11 (70)^abc^	17.30 ± 0.13 (50)^abc^
	Muscle	32.90 ± 0.22 (100)	20.07 ± 0.13 (61)^abc^	14.48 ± 0.12 (44)^abc^

Footnotes are same as given in Table 2.
